# Disruption of action potential and calcium signaling properties in malformed myofibers from dystrophin-deficient mice

**DOI:** 10.14814/phy2.12366

**Published:** 2015-04-23

**Authors:** Erick O Hernández-Ochoa, Stephen J P Pratt, Karla P Garcia-Pelagio, Martin F Schneider, Richard M Lovering

**Affiliations:** 1 Department of Biochemistry & Molecular Biology, University of Maryland School of MedicineBaltimore, Maryland; 2 Department of Orthopaedics, University of Maryland School of MedicineBaltimore, Maryland; 3 Department of Physiology, University of Maryland School of MedicineBaltimore, Maryland

**Keywords:** Action potential, animal model of muscular dystrophy, Ca^2+^ indicator, Ca^2+^ transients, di-8-ANEPPS, Duchenne muscular dystrophy, elastimetry, MDX, myofiber branching, sarcolemma biomechanics, T-tubule morphology, voltage-sensitive dye

## Abstract

Duchenne muscular dystrophy (DMD), the most common and severe muscular dystrophy, is caused by the absence of dystrophin. Muscle weakness and fragility (i.e., increased susceptibility to damage) are presumably due to structural instability of the myofiber cytoskeleton, but recent studies suggest that the increased presence of malformed/branched myofibers in dystrophic muscle may also play a role. We have previously studied myofiber morphology in healthy wild-type (WT) and dystrophic (MDX) skeletal muscle. Here, we examined myofiber excitability using high-speed confocal microscopy and the voltage-sensitive indicator di-8-butyl-amino-naphthyl-ethylene-pyridinium-propyl-sulfonate (di-8-ANEPPS) to assess the action potential (AP) properties. We also examined AP-induced Ca^2+^ transients using high-speed confocal microscopy with rhod-2, and assessed sarcolemma fragility using elastimetry. AP recordings showed an increased width and time to peak in malformed MDX myofibers compared to normal myofibers from both WT and MDX, but no significant change in AP amplitude. Malformed MDX myofibers also exhibited reduced AP-induced Ca^2+^ transients, with a further Ca^2+^ transient reduction in the branches of malformed MDX myofibers. Mechanical studies indicated an increased sarcolemma deformability and instability in malformed MDX myofibers. The data suggest that malformed myofibers are functionally different from myofibers with normal morphology. The differences seen in AP properties and Ca^2+^ signals suggest changes in excitability and remodeling of the global Ca^2+^ signal, both of which could underlie reported weakness in dystrophic muscle. The biomechanical changes in the sarcolemma support the notion that malformed myofibers are more susceptible to damage. The high prevalence of malformed myofibers in dystrophic muscle may contribute to the progressive strength loss and fragility seen in dystrophic muscles.

## Introduction

The most common and severe form of muscular dystrophy is Duchenne muscular dystrophy (DMD), a disorder caused by the absence of dystrophin, a structural protein found on the cytoplasmic surface of the sarcolemma. The MDX mouse also lacks dystrophin and has been widely used as an animal model of DMD (Willmann et al. 2009). While the exact role of dystrophin remains unclear, its absence in DMD results in significant myopathy. Because of the increased susceptibility to damage, patients with DMD are typically advised to avoid resistive exercise and yet, still have progressive muscle pathology, such as muscle fiber (myofiber) degeneration, fibrosis, and inflammation. Although the absence of dystrophin remains constant but the disease is progressive, a secondary cumulative effect is indicated. Several studies suggest that the age-dependent increase in the number of malformed myofibers within the muscle contributes to a decrease in muscle-specific force (Head et al. 1990) and an increase in susceptibility to contraction-induced injury (Chan et al. 2007).

**Figure 1:**
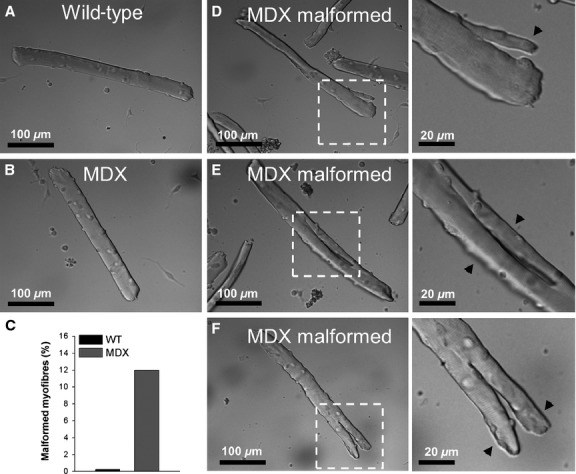
Typical morphology of malformed MDX myofibers. Representative differential interphase contrast microscopy images of wild-type (A), MDX (B), and malformed MDX (D–F) flexor digitorum brevis (FDB) fibers. Panel C shows quantification (%) of malformed myofibers in wild-type and MDX FDB muscle. *Left panels* in D–F show MDX-malformed myofibers with a process (D), a split (E), and with a bifurcation (F). *Right panels* in D–F are magnified versions of dash-boxed regions indicated in left panels. The arrows indicate branches.

Myofibers in skeletal muscle are typically long and cylindrical in shape (Fig.1A, B), but can also be malformed, with a variety of branching patterns (Fig.1D–F). It is well documented that malformed myofibers are not simply structural associations between two different cells, but instead have branches that are continuous strands from a single myofiber (Head et al. 1990; Head 2012). The genesis and persistence of myofiber malformations are still unclear, but they have been identified in diseased (Head et al. 1990; Chan et al. 2007), regenerating (Schmalbruch 1976), and hypertrophied muscle (Snow and Chortkoff 1987; Eriksson et al. 2006); therefore the mechanism responsible for the morphological alteration in MDX muscle is not specific to the lack of dystrophin. In dystrophic muscle, it is plausible that this morphological anomaly arises due to disruptions in the muscle growth/regeneration program (Snow and Chortkoff 1987; Tamaki et al. 1993; Head 2012).

Muscle weakness and fragility are hallmarks of dystrophic muscle. If malformed myofibers do indeed contribute to these parameters, then recognizing how they differ from myofibers with normal morphology is paramount to understanding dystrophic muscle. There are many differences between healthy and dystrophic myofibers (Petrof 1998; Woods et al. 2004; Friedrich et al. 2008; Hollingworth et al. 2008; Khairallah et al. 2012; Lovering et al. 2013; Pratt et al. 2013), and some recent studies suggest that malformed myofibers are a subset of dystrophic myofibers that are further altered, with distorted microarchitecture (Friedrich et al. 2010; Buttgereit et al. 2013), increased susceptibility to damage (Chan et al. 2007), and further impaired sarcolemma strength (Head 2010).

We have previously shown alterations in Ca^2+^ handling in malformed myofibers (Lovering et al. 2009; Goodall et al. 2012). The aim of this study was to provide further insight into excitation–contraction coupling and sarcolemma mechanics of malformed MDX myofibers. We tested the hypothesis that both action potential and calcium signaling properties are disrupted in malformed myofibers from the MDX mouse compared to myofibers with normal morphology. We also tested whether the membrane of the myofiber, the sarcolemma, was mechanically different in malformed myofibers. Since there is an age-dependent increase in malformed myofibers (Chan et al. 2007; Lovering et al. 2009), the combination of altered contractile activation with increased mechanical instability could help explain the progressive fragility of dystrophic muscle. Finally, in addition to comparisons between healthy wild-type (WT), MDX, and MDX-malformed myofibers, we also searched for differences *within* the malformed myofibers (i.e., trunk vs. branch). The results support the notion that MDX-malformed myofibers are functionally different from MDX myofibers with normal morphology.

## Methods

### Animals and myofiber preparation

We used age-matched male control mice (wild-type, WT) and MDX mice (lacking dystrophin) from the C57BL/10ScSnJ strain (The Jackson Laboratory, Bar Harbor, ME). A total of 20 mice were used (approximately 3–4 months of age) and all experimental procedures were approved by the University of Maryland Institutional Animal Care & Use Committee.

Following euthanasia (CO_2_ inhalation), flexor digitorum brevis (FDB) muscles were harvested bilaterally from MDX and WT mice. Single myofibers were enzymatically isolated in DMEM with 0.2% bovine serum albumin (BSA, Sigma, St. Louis, MO, A7906), 1 *μ*L/mL Gentamicin (Sigma, G1397), and 2 mg/mL type I collagenase (Sigma, C0130) for 1–3 h at 37°C as previously described (Brown et al. 2007; Cherednichenko et al. 2008). Solutions were filtered using a 0.2 *μ*m polyethersulfone membrane (Thermo, 194–2520). Myofibers were then plated on extracellular matrix-coated (ECM, Sigma, E1270) imaging dishes (P35G-1.0-14-C, Matek Inc.) and rested for 12 h before experiments. All of the myofibers were imaged and/or tested within a 24-h period, thus avoiding changes that can occur in FDB myofibers that are cultured for prolonged periods (Ravenscroft et al. 2007).

### Transverse-tubular network imaging in living myofibers

Wild-type and MDX myofibers were stained with the voltage-sensitive dye pyridinium, 4-[2-(6-(dioctylamino)-2-naphthalenyl) ethenyl]-1-(3-sulfopropyl)-, inner salt (di-8-ANEPPS; Life Technologies, Carlsbad, CA, Cat. No. D3167). Myofibers were incubated with di-8-ANEPPS (2.5 *μ*mol/L per L in DMEM media) for 3 h at 37°C, washed in L-15 media plus 2.5 *μ*mol/L di-8-ANEPPS, and then imaged on a Fluoview 500 confocal system (Olympus; ×60, 1.3 NA water-immersion objective; pixel dimensions 0.2 × 0.2 *μ*m in *x* and *y*) using L-15 media. Confocal images (512 × 512 pixels) of the tubular network were obtained from randomly selected myofibers using the same image acquisition settings and enhancing parameters. All malformed myofibers observed were used for imaging. Images were background corrected and a region of interest (ROI) of fixed dimensions was used to estimate the average fluorescence profile within the region of interest.

### Action potential recordings

Potentiometric dye action potential (AP) recordings and analysis were performed as previously described (Prosser et al. 2010; Hernandez-Ochoa et al. 2012). FDB myofibers were stained with 2.5 *μ*mol/L di-8-ANEPPS in the incubator for 3 h, followed by three washes in L-15 media. Myofiber cultures were mounted on a Zeiss LSM 5 LIVE high-speed confocal system (Carl Zeiss, Jena, Germany) and stimulated with dual platinum field electrodes. Individual myofibers were imaged with a 60×/1.3 NA water-immersion objective lens. Myofiber fluorescence was excited with a 532-nm diode laser, and fluorescence emission above 550 nm was sampled during repeated line scans through the interior of myofibers (100 *μ*s/line). The line scan was conducted at a depth of ∼15–20 *μ*m into the interior of the myofiber. One millisecond electrical field stimuli were applied via two parallel platinum wires positioned at the bottom of the dish, ∼5 mm apart, to elicit action potentials (myofibers were centrally positioned so that electrodes were equidistant). Application of each stimulation protocol was synchronized relative to the start of confocal scan acquisition. Typically, the field stimulus was applied 100 ms after the start of the confocal scan sequence, thus providing control images before stimulation at the start of each sequence. These control images were used to determine the resting steady-state fluorescence level (F0). Average intensity of fluorescence within selected ROIs was measured with Zeiss LSM Image Examiner. Line scan (x-t) images (frame size: 512 × 10,000 lines; scan speed: 100 *μ*s/line for 1 s acquisition) were background corrected by subtracting an average value recorded outside the cell. The average F0 value in each ROI before electrical stimulation was used to scale di-8-ANEPPS signals in the same ROI as ΔF/F0. Previous studies in cultured myofibers have shown that di-8-ANEPPS signals exhibit temporal properties virtually identical to those of electrical recordings of the AP (DiFranco et al. 2005; Prosser et al. 2010). Signals were converted to −ΔF/F0 values, and four trials with the same electrode polarity were averaged to increase the signal-to-noise ratio. All single myofiber recordings were performed at room temperature, 21–23°C.

### Rhod-2 high-speed confocal Ca^2+^ imaging

Rhod-2 measurements were carried out on a high-speed confocal system (LSM 5 Live) as previously described (Prosser et al. 2010; Hernandez-Ochoa et al. 2012). Myofibers were loaded with 1 *μ*mol/L rhod-2 AM (Life Technologies, Carlsbad, CA, Cat. No. R1245MP) in L-15 media supplemented with 0.25% w/v BSA for 1 h at room temperature. Then the myofibers were washed once with L-15 media for 5 min prior to beginning recording to remove residual rhod-2AM. Individual myofibers were imaged with a 60×/1.3 NA water-immersion objective lens. Excitation for rhod-2 was provided by the 532-nm line of a 100-mW diode laser, and emitted light was collected at >550 nm. Action potential-induced Ca^2+^ transients were triggered using the same 1-ms electrical stimulus as in di-8-ANEPPS assays. Application of each stimulation protocol was synchronized relative to the start of confocal scan acquisition. Typically, the field stimulus was applied 100 ms after the start of the confocal scan sequence, thus providing control images before stimulation at the start of each sequence. These control images were used to determine the resting steady-state fluorescence level (F0). Average intensity of fluorescence within selected ROIs was measured with Zeiss LSM Image Examiner. Images in line scan (x-t) mode (frame size: 512 × 10,000 pixels; scan speed: 100 *μ*s/line for 1 s acquisition) were background corrected by subtracting an average value recorded outside the cell. The average F0 value in each ROI before electrical stimulation was used to scale Ca^2+^ signals in the same ROI as ΔF/F0. No attempts were made to estimate the actual cytosolic Ca^2+^ concentration. Ca^2+^ imaging experiments were carried out at room temperature, 21–23°C. Only myofibers responding to alternate polarities were included in the analysis of AP and AP-evoked Ca^2+^ transients.

### Elastimetry

To assess whether malformed myofibers have altered mechanical properties, we used a recently established method (Garcia-Pelagio et al. 2011) to compare sarcolemma membrane fragility on enzymatically dissociated myofibers (both normal and malformed) from the FDBs of WT and MDX mice. For these studies, isolated myofibers were plated on ECM-coated (Sigma E1270) imaging dishes (Matek, P35G-1.0-14-C), transferred to an experimental chamber containing DMEM media supplemented with 0.2% fetal bovine serum (FBS), and fixed to a stage of a compound Laborlux microscope (E. Leica Microsystems, Wetzlar, Germany). The optical part of the microscope was placed on an XY stage so that every part of the muscle cell could be visualized without disturbing the preparation or the subsequent placement of a glass micropipette. Photomicrographs were taken with a digital camera (SONY *α*-330; Sony Corporation, Tokyo, Japan) through a 10× eyepiece and 25× or 40×, N.A. 0.75 water-immersion objective. Sarcolemma bleb displacement and diameter of the myofiber were measured with Image J software (NIH, Bethesda, MD). Experiments were performed at 30°C, controlled by a GB32J36 thermistor (Fenwal Electronics, Framingham, MA), connected to a temperature controller (Yellow Springs Instrument Co., Inc, Ohio OH), which maintains the temperature through a two Peltier modulus (Midland Ross, Cambridge, MA). A micropipette, attached to manometers, was placed on the surface of the myofiber, and a bleb was formed as negative suction pressure (P) was applied to assess the elastic behavior of the sarcolemma. We analyzed the elastic behavior of the distortion and tension lines formed by myofibrils, and the membrane in response to suction pressures (P) applied over a small area as a bleb is formed, as previously reported (Garcia-Pelagio et al. 2015). Pressure exerted to the outside surface of the myofiber membrane was calculated from P = *ρg*h_man_ in dynes/cm^2^, where *ρ *= manometer's fluid density in g/cm^3^, *g *=* *981 cm/s^2^, and h is the difference of levels in the manometer (cm) following the hydrostatic pressure equation. After bleb formation, continuous pressure was applied until rupture of the cell membrane (membrane bursting). Maximal pressure needed to cause disruption (P_burst_) was recorded and used to compare sarcolemma stability between groups.

### Statistical analysis

All data processing and statistical analysis was performed using OriginPro 8.0 (OriginLab, Northhampton, MA). All data are presented as mean ± SE unless otherwise noted. Statistical significance was assessed using either the parametric two sample *t*-test or with the nonparametric Mann–Whitney rank-sum test. Significance was set at *P *<* *0.05.

## Results

### Myofiber morphology

Malformed myofibers are much more prevalent in MDX muscle than in WT muscle (Head et al. 1992; Lovering et al. 2009; Friedrich et al. 2010). Here, we used the flexor digitorum brevis muscle (FDB) for our experiments and we describe the number and type of malformed myofibers observed after single fiber isolation (Fig.1). The percent of malformed myofibers (11.61%) in the MDX muscles made locating them straightforward, whereas finding malformed myofibers in WT muscle (0.23%) is extremely difficult (Fig.1C) (Lovering et al. 2009). There was a variety of branching patterns in MDX-malformed myofibers (Fig.1) and the number and type of malformed myofibers observed were similar to those previously described (Lovering et al. 2009; Chan and Head 2011; Goodall et al. 2012).

### T-tubule morphology

**Figure 2:**
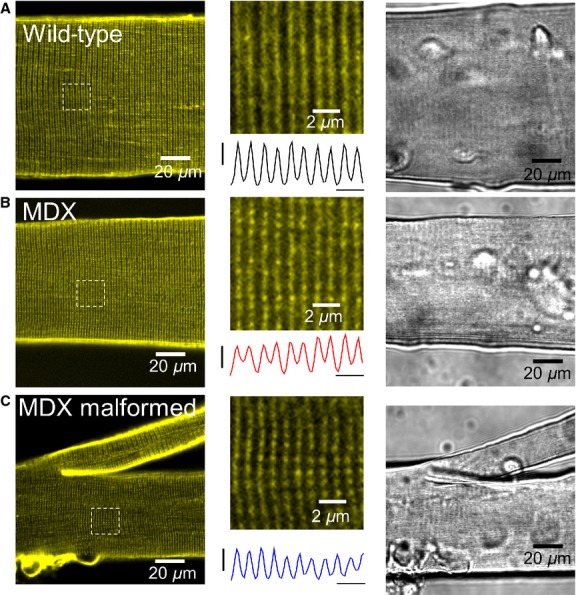
Confocal microscopy and differential interference contrast (DIC) images of wild-type and MDX myofibers stained with di-8-ANEPPS. A–C, *left panels*: confocal images of FDB myofibers from wild-type (A) and MDX (B, C) mice. Myofibers were cultured for 12 h and then stained with di-8-ANEPPS. *Middle panels*: zoomed-in versions of boxed regions, as indicated in left panels. Traces below show averaged di-8-ANEPPS fluorescence profiles across the boxed region, horizontal scale bar: 2 *μ*m. T-tubules are organized in a regular striated pattern, characterized by a ∼2-*μ*m sarcomere length, and ∼1 *μ*m T-tubule spacing. No changes in T-tubule morphology are seen in MDX myofibers, both unbranched and malformed, when compared to wild-type. *Right panels*: DIC images from the same myofibers illustrated in left panels.

The sarcolemma and the transverse-tubules are the membrane systems that allow membrane depolarization to spread both longitudinally and inwardly into the myofiber, respectively (Franzini-Armstrong and Porter 1964; Franzini-Armstrong and Jorgensen 1994). Disruption or loss of continuity of these systems would affect the propagation of the action potential and thus the contraction of the myofiber. To examine the integrity of the T-tubule system, FDBs from WT and MDX mice were cultured and stained with di-8-ANEPPS dye (Fig.2). T-tubules were organized in a typical striated pattern, characterized by a ∼2-*μ*m sarcomere length and ∼1 *μ*m T-tubule spacing. Similar to gross examination of the cytoskeleton (Lovering et al. 2009; Goodall et al. 2012), no changes in T-tubule morphology were seen in MDX myofibers, normal or malformed, when compared to WT.

### Action potential measurements

In skeletal muscle the AP, via sequential activation of the voltage sensors of voltage-gated Ca^2+^ channels (Cav1.1) and the mechanically coupled Ca^2+^ release channels (RyR1), triggers Ca^2+^ release from the sarcoplasmic reticulum (SR), and ultimately muscle contraction, in a process known as excitation–contraction (E–C) coupling (Schneider and Chandler 1973; Rios and Brum 1987; Schneider and Hernandez-Ochoa 2012). We have previously shown alterations in AP-induced Ca^2+^ transients in malformed myofibers (Lovering et al. 2009; Goodall et al. 2012). Alterations in AP properties could explain the depressed Ca^2+^ transients in MDX myofibers with normal morphology and in MDX-malformed myofibers.

**Figure 3:**
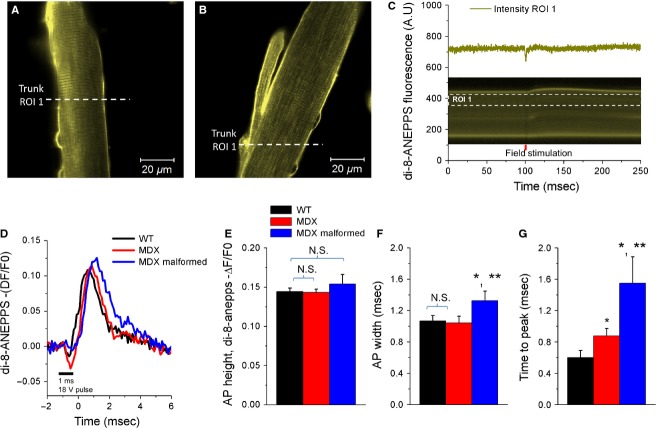
Action potential time to peak and rising phase is increased in malformed MDX myofibers. Representative confocal *x-y* images of a wild-type myofiber (A) and a malformed MDX myofiber (B) stained with the voltage-sensitive dye di-8-ANEPPS. Dashed lines in *A* and *B* indicate the region of interest (ROI) of the line scan used to measure action potentials in the cytoplasm (trunk, ROI 1) of normal WT and MDX myofibers or in the trunk (ROI 1) of malformed MDX myofibers. C, *bottom*: line scan image from ROI indicated in A. C, *top*: time course of di-8-ANEPPS fluorescence in response to single field stimulation measured in the region indicated by white dashed box. D, average change in di-8-ANEPPS fluorescence, reported as −ΔF/F0, in wild-type (black trace), normal MDX (red trace), and malformed MDX (blue trace) FDB myofibers in response to field stimulation. E–G, summary of action potential properties in WT (black bars), MDX (red bars), and malformed MDX (blue bars) FDB myofibers. No significant change in action potential height was found between groups (*P* > 0.05, WT: *n* = 8, MDX: *n* = 14; MDX-malformed: *n* = 10). MDX-malformed myofibers demonstrated a significant increase in action potential width and time to peak compared wild-type and MDX fibers with normal morphology (*P* < 0.05; WT: *n* = 8, MDX-malformed *n* = 14; MDX-malformed *n* = 10). *indicates *P* < 0.05 compared to wild-type, **indicates *P* < 0.05 compared to MDX, using two sample *t*-test.

To evaluate any modifications of the propagated AP, here we measured AP properties using the potentiometric dye di-8-ANEPPS. We determined the response of myofibers to electrical stimulation by rapidly acquiring a line scan image (x-t image; 100 *μ*s/line) in a continuous fashion, before, during, and after stimulation, of a line across the myofiber, and then quantifying the di-8-ANEPPS fluorescence (Fig.3A–C). We were able to discern temporal differences in the AP properties between WT, MDX, and MDX-malformed myofibers, as depicted in Figure3. Optical single cell di-8-ANEPPS recordings showed that the action potential width and time to peak are significantly increased in malformed MDX myofibers (Fig.3D, F, G). The AP width was prolonged by 24.2% in MDX-malformed myofibers compared with WT, as quantified in Figure3F. The time to peak was also increased in MDX-malformed myofibers to 1.5 ms, compared with 0.6 ms for WT, corresponding to a 158.3% increase in AP time to peak (Fig.3G). Despite the significant increase in AP width and time to peak in MDX-malformed myofibers, when compared to the WT and MDX normal morphology counterparts, there was no significant change in action potential height (−ΔF/F0) between groups (Fig.3D, E; WT: 0.14 ± 0.01; MDX: 0.14 ± 0.01; MDX malformed: 0.15 ± 0.03, *P* > 0.05). Taken together, these results suggest that MDX malformed myofibers exhibit kinetic alterations on AP properties.

**Figure 4:**
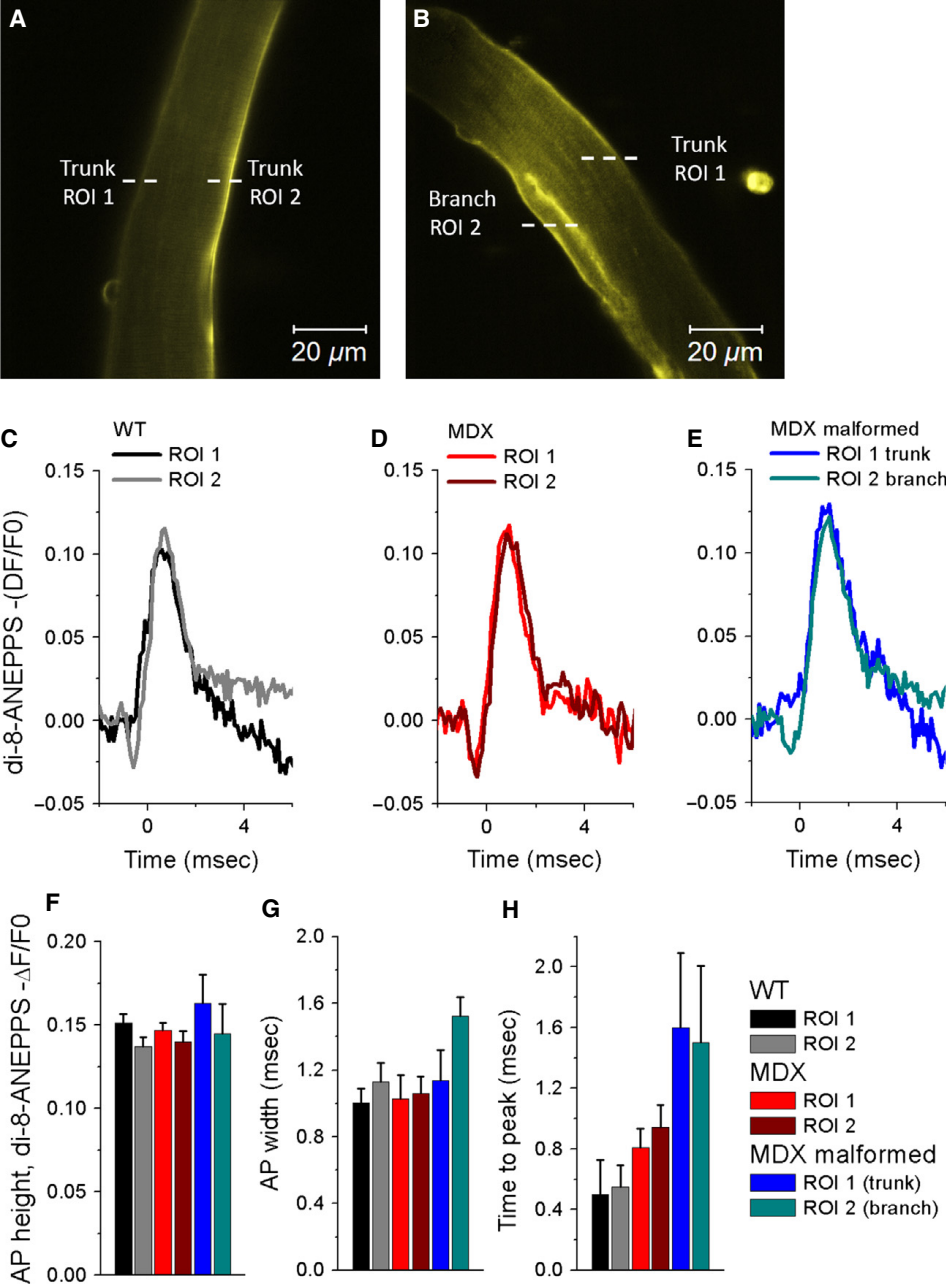
The action potential properties are indistinct in the trunk or in the branch of malformed MDX myofibers. Representative confocal *x-y* images of a wild-type (A) myofiber and a MDX malformed myofiber (B) stained with the voltage-sensitive dye di-8-ANEPPS. White dashed lines in *A* and *B* indicate the regions of interest (ROI) of the line scan used to measure action potentials in the cytoplasm (trunk, ROI 1 and ROI 2) of normal WT and MDX myofibers or in the trunk (ROI 1) and branch (ROI 2) of malformed MDX myofibers. Average change in di-8-ANEPPS fluorescence in FDB myofibers in response to field stimulation, reported as −ΔF/F0 and measured in ROIs of the line scan, was used to measure action potentials in the two regions of interest (ROIs, ROI 1, and ROI 2 as illustrated in A–B) in the trunk of wild-type myofibers (C; ROI 1, black trace; ROI 2 gray trace), MDX myofibers (D; ROI 1, red trace; ROI 2, dark red trace) or in the trunk (ROI 1, blue trace) and in the branch (ROI 2, dark cyan trace) of malformed MDX myofibers (E). F–H, summary of action potential properties measured in two ROIs in WT (black and gray bars), MDX (red and dark red bars), and MDX malformed (blue and dark cyan bars) FDB myofibers. No significant change in AP height, width, or time to peak was found between two ROIs from the trunk of wild-type, MDX myofibers, nor between two ROIs (one from the trunk and another from the branch) of malformed MDX myofibers (*P* > 0.05, WT: *n* = 8, MDX unbranched *n* = 14; MDX branched *n* = 10). *P* > 0.05 RO1 1 versus ROI 2, using two sample *t*-test.

To further investigate excitability within the different branching areas of MDX malformed myofibers, we compared action potential properties in the trunk versus branch of malformed myofibers (Fig.4, ROI 1 and ROI 2, respectively). The data show that the action potential properties were no different when comparing signals in the trunk or in the branch of malformed MDX myofibers (Fig.4E–H). No significant differences were found in the AP peak (−ΔF/F0) (WT: ROI 1 = 0.15 ± 0.005, ROI 2 = 0.13 ± 0.005; MDX: ROI 1 = 0.14 ± 0.004, ROI 2 = 0.13 ± 0.006; MDX malformed: ROI 1 = 0.16 ± 0.016, ROI 2 = 0.14 ± 0.017), AP width (ms) (WT: ROI 1 = 1.0 ± 0.08, ROI 2 = 1.13 ± 0.11; MDX: ROI 1 = 1.0 ± 0.14, ROI 2 = 1.0 ± 0.10; MDX malformed: ROI 1 = 1.1 ± 0.18, ROI 2 = 1.5 ± 0.11) and AP time to peak (ms) (WT: ROI 1 = 0.5 ± 0.22, ROI 2 = 0.5 ± 0.14; MDX: ROI 1 = 0.8 ± 0.12, ROI 2 = 0.9 ± 0.14; MDX malformed: ROI 1 = 1.6 ± 0.49, ROI 2 = 1.5 ± 0.50; *P* > 0.05).

### Action potential-induced Ca^2+^ transients

**Figure 5:**
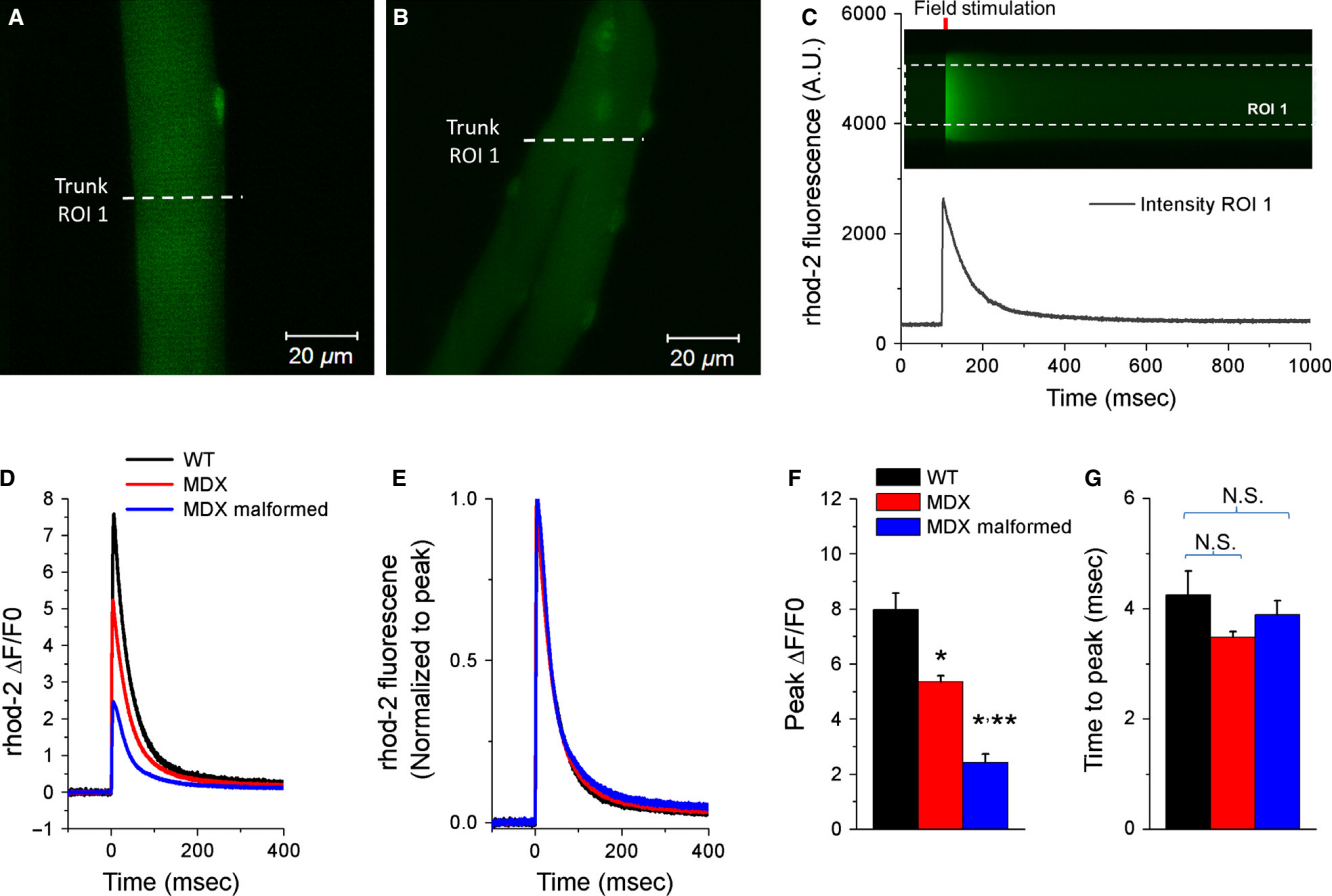
MDX myofibers exhibit reduced action potential-induced Ca^2+^ transients. Flexor digitorum brevis (FDB) myofibers were isolated from MDX and wild-type mice and then loaded with the Ca^2+^-sensitive dye rhod-2 and their calcium responses to electrical stimulation were recorded using high-speed confocal microscopy. Representative confocal *x-y* images of a wild-type myofiber (A) and a MDX malformed myofiber (B) loaded with rhod-2. White dashed lines in *A* and *B* indicate examples of the region of interest (ROI) of the line scan used to measure action potential-induced Ca^2+^ transient in the cytoplasm (trunk, ROI 1) of normal WT and MDX myofibers or in the trunk (ROI 1) of malformed MDX myofibers. C, *top*: line scan (*x*-*t*) image from ROI indicated in A. C, *bottom*: time course of rhod-2 fluorescence in response to single field stimulation measured in the region indicated by white dashed box in C *top*. D, average change in rhod-2 fluorescence, reported as ΔF/F0, in wild-type (black trace), MDX (red trace), and MDX malformed (blue trace) FDB myofibers in response to field stimulation. E, traces from *D* normalized to peak transient amplitude. F–G, summary of action potential-induced Ca^2+^ transient properties in WT (black bars), MDX (red bars), and MDX malformed (blue bars) FDB myofibers. F, a significant reduction in electrically evoked Ca^2+^ transient peak was found in MDX myofibers when compared to WT counterparts. MDX malformed myofibers displayed a more profound reduction on the amplitude of the Ca^2+^ transient (*P* < 0.05, WT: *n* = 10, MDX 16; MDX malformed 14). G, no significant change in Ca^2+^ transient time to peak was found between groups. *indicates *P* < 0.05 compared to wild-type, **indicates *P* < 0.05 compared to MDX, using two sample *t*-test.

Our previous reports evaluated Ca^2+^ transients in MDX myofibers elicited by a single AP using a relatively low-temporal resolution and low signal-to-noise ratio Ca^2+^ imaging system (Lovering et al. 2009; Goodall et al. 2012). Here, we sought to build on this work by evaluating action potential-induced Ca^2+^ transients using a high-speed, high signal-to-noise confocal microscopy system. To assess calcium responses to stimulation, FDB myofibers were isolated from MDX and WT mice and then loaded with the Ca^2+^-sensitive dye rhod-2. AP-induced Ca^2+^ transients were triggered using the same electrical stimulus as in the di-8-ANEPPS assays and fluorescence signals recorded using the high-speed and high-sensitivity confocal imaging system (100 *μ*s/line). MDX myofibers exhibited reduced action potential-induced Ca^2+^ transients (Fig.5D, F) from WT myofibers, and malformed MDX myofibers showed a further reduction in Ca^2+^ transients from MDX myofibers with normal morphology. As quantified in Fig.5F, MDX and MDX malformed myofibers exhibit a 32.8% and 69.6% decrease in peak ΔF/F0, respectively, when compared with WT following single AP stimulation (WT: 7.98 ± 0.59; MDX: 5.36 ± 0.21, *P* < 0.05 vs. WT; MDX malformed: 2.42 ± 0.29, *P* < 0.05 vs. WT). Because resting myoplasmic Ca^2+^ concentration is similar in WT and MDX myofibers (Lovering et al. 2009; Goodall et al. 2012), and as ΔF/F0 records correct for differences in dye loading, these values represent differences in the Ca^2+^ transients between WT and MDX myofibers. The above results further demonstrate that MDX myofibers, both normal and malformed, exhibit alterations in Ca^2+^ release following electrical stimulation. The time to peak of Ca^2+^ release from the SR internal store following electrical excitation can be indirectly monitored by evaluating the time to peak of the rising phase of the Ca^2+^ transient. We were unable to discern differences in the time to peak of Ca^2+^ release between WT and MDX myofibers, as depicted in Fig.5G. Taken together, these results suggest that the lack of dystrophin affects the amplitude of Ca^2+^ transient, but not its time course in fast-twitch myofibers.

**Figure 6:**
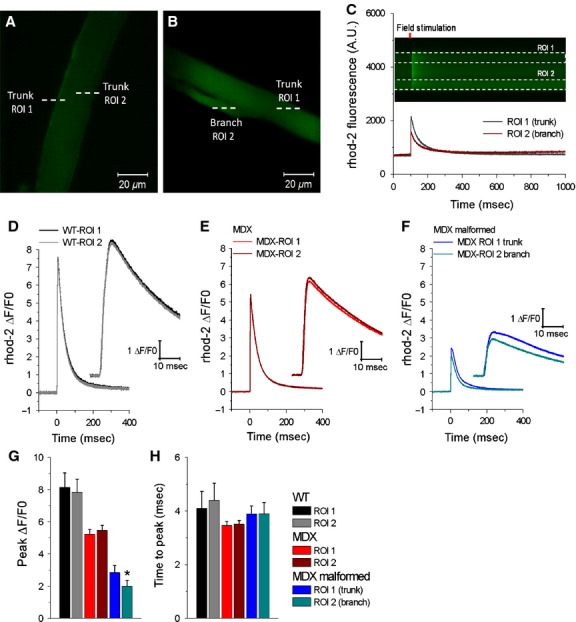
Action potential-induced Ca^2+^ transients in branched segments are more depressed compared to the trunk segments of malformed MDX myofibers. Representative confocal *x-y* images of a WT myofiber (A) and a malformed MDX myofiber (B) loaded with rhod-2. White dashed lines in A and B indicate examples of regions of interest (ROIs) of the line scan used to measure action potential-induced Ca^2+^ transients in the cytoplasm (trunk, ROI 1 and ROI 2) of normal WT and MDX myofibers or in the trunk (ROI 1) and branch (ROI 2) of malformed MDX myofibers. C, *top*: line scan (x-t) image from ROIs indicated in malformed MDX myofiber in B. C, *bottom*: time course of rhod-2 fluorescence in response to single field stimulation measured in the regions indicated by white dashed boxes in C top. The amplitude of the Ca^2+^ transient is reduced in the branch when compared to trunk segment of the malformed MDX myofiber. D–F, Average change in rhod-2 fluorescence in FDB myofibers in response to field stimulation, measured in two regions of interest in the trunk of wild-type myofibers (D; ROI 1, black trace; ROI 2 gray trace), MDX myofibers (E; ROI 1, red trace; ROI 2, dark red trace) or in the trunk (ROI 1, blue trace) and in the branch (ROI 2, dark cyan trace) of malformed MDX myofibers (F). Insets in D–F, show a time expanded-versions of the rising phase of the Ca^2+^ transients from panels D–F. G–H, Summary of action potential-induced Ca^2+^ transient properties in WT (black and gray bars), MDX (red and dark red bars), and MDX malformed (blue and cyan bars) FDB myofibers evaluated at two regions within the myofibers (different ROIs). No differences in electrically evoked Ca^2+^ transient peak measured at two ROIs were found between WT and MDX myofibers with normal morphology. MDX malformed myofibers demonstrated a small but significant decrease in the amplitude of action potential-induced Ca^2+^ transients from branches when compared to signals measured in the trunk of MDX malformed myofibers (*P* < 0.05, WT: *n* = 10, MDX 16; MDX malformed 14). No significant change in action potential-induced Ca^2+^ transient time to peak was found in two different regions of interest (ROI 1 and ROI 2) between groups. *indicates *P* < 0.05 between ROI 1 and ROI 2 using two sample *t*-test.

To further investigate excitability within the MDX malformed myofibers, we compared AP-induced Ca^2+^ transients’ properties in the trunk versus branch of malformed myofibers (Fig.6, ROI 1 and ROI 2, respectively). The findings show a significant reduction in the amplitude of the AP-induced Ca^2+^ transients in the branched segments when compared to the trunk segments of malformed MDX myofibers (Fig.6F, G). Figure6G shows pooled data of AP-induced Ca^2+^ transient properties from two trunk regions (ROI 1 and ROI 2) in WT and MDX myofibers, and from the trunk (ROI 1) and branched segments (ROI 2) of MDX malformed myofibers (ΔF/F0 peak amplitude: WT: ROI 1 = 8.1 ± 0.9, ROI 2 = 7.8 ± 0.8, *P* > 0.05; MDX: ROI 1 = 5.2 ± 0.2, ROI 2 = 5.4 ± 0.3, *P* > 0.05; MDX malformed: ROI 1 = 2.8 ± 0.4, ROI 2 = 1.9 ± 0.3; *P* < 0.05). No significant differences were found in the time to peak (ms) (WT: ROI 1 = 4.1 ± 0.6, ROI 2 = 4.4 ± 0.6, *P* > 0.05; MDX: ROI 1 = 3.4 ± 0.1, ROI 2 = 3.5 ± 0.1, *P* > 0.05; MDX malformed: ROI 1 = 3.8 ± 0.3, ROI 2 = 3.8 ± 0.4, *P* > 0.05). These findings suggest that there are asymmetries in the generation of AP-induced Ca^2+^ transients in the branched segment when compared to trunk segment of MDX malformed myofibers.

### Biomechanics of the surface sarcolemma of WT, MDX, and malformed MDX myofibers

**Figure 7:**
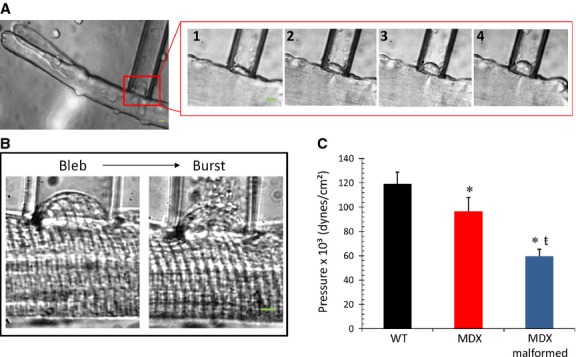
Increased sarcolemma deformability and instability in MDX malformed myofibers. A: Example of a bleb forming inside a pipette on the trunk of a MDX malformed myofiber. Boxed region shows sarcolemma bleb increasing in size (1–4) with increasing pressure, (250× magnification). B: Increasing negative pressure was applied to the sarcolemma membrane until the surface membrane rupture (burst, P_burst_); example of higher power image (400×) showing the bleb and burst. C: Histogram of the membrane bursting data. Maximal pressure needed to cause disruption (P_burst_) was reduced in MDX myofibers and further reduced in myofibers with altered morphology. Data presented as mean ± SD, *P* < 0.05. *indicates significant difference from WT (sample size: WT = 12, MDX = 7, MDX malformed = 8). ŧ indicates significant difference from MDX. Scale bar = 5 *μ*m.

To study the biomechanical properties of the sarcolemma, suction pressures (P) were applied through a micropipette to the myofiber membrane to generate a bleb (Fig.7A 1–4), which increased in height with increasing P (Garcia-Pelagio et al. 2015). Larger increases in P ruptured the connections between the sarcolemma and myofibrils and eventually caused the sarcolemma to burst (Fig.7B). Compared to healthy WT myofibers, the pressure required to induce sarcolemma bursts (P_burst_) was significantly lower (19%) in MDX myofibers and even less (50%) in malformed MDX myofibers (Fig.7C). To further investigate mechanical stability within the MDX malformed myofibers, we compared sarcolemma properties in the trunk versus branch of malformed myofibers. The data indicate no further difference in P_burst_ between the trunk and the branch of malformed MDX myofibers (not shown). Overall, the mechanical data indicate an increase in sarcolemma deformability and instability in MDX muscle. These parameters were further exacerbated in malformed myofibers.

## Discussion

The genetic basis for DMD has been determined (Hoffman et al. 1987; Wagner 2002; Lovering et al. 2005; McNally and Pytel 2007), but the mechanisms responsible for the decrease in muscle-specific force (force normalized to muscle cross-sectional area) and increased susceptibility to injury are still being clarified. Hypotheses for the heightened loss of force after injury include structural weakness of the myofiber cytoskeleton, or changes in signaling secondary to the loss of dystrophin (Lovering et al. 2009). It is unlikely that any one finding can account for the totality of functional changes in damaged muscle, however, an additional complexity has been brought to light that appears to contribute to the dystrophic phenotype; the increased presence of malformed myofibers (Chan and Head 2011; Faber et al. 2014). The incidence of malformed myofibers is now well documented in dystrophic muscle but is extremely low in healthy wild-type muscle (0%, Lovering et al. 2009), thus malformed wild-type myofibers would be difficult to include in studies such as these, as the number of animals sacrificed to find enough of them would be unjustified.

It is possible to confirm the presence of a true myofiber split from thin serial sections of whole muscle (Isaacs et al. 1973; Schmalbruch 1984), but a full reconstitution of morphology for each myofiber requires tedious serial sectioning throughout the whole muscle. In this investigation, we used enzymatically dissociated myofibers from the FDB. We were able to see the overall morphology of malformed MDX myofibers with light microscopy and confirm an increased number of malformed myofibers in dystrophic muscle. Staining with di-8-ANEPPS allowed us to assess greater detail of intracellular architecture, showing that the T-tubule structure was quite similar to the normal WT myofibers and to MDX myofibers where no malformations were identified. Despite an appearance of a normal T-tubule structure seen here and the previously described normal cytoskeletal structure (Lovering et al. 2009), eloquent studies have been performed to show that the microarchitecture within malformed myofibers is significantly altered (Friedrich et al. 2010; Buttgereit et al. 2013).

Excitation–contraction coupling (E–C coupling) is operationally defined as the sequence of events from propagation of the action potential along the sarcolemma to the release of Ca^2+^ from the sarcoplasmic reticulum (SR), a process through which neural activation results in a muscle contraction. Our results show no changes in the amplitude of the AP height between groups, but AP width and time to peak were significantly increased in malformed MDX myofibers compared to WT and MDX myofibers with normal morphology (Fig.3D, F, G). We also demonstrate significant differences in the AP-evoked Ca^2+^ release in malformed MDX myofibers when compared to normal WT and MDX myofibers, and a further reduction in the branched portion of the myofiber when compared to the trunk. Results of the sarcolemma mechanics showing increased weakness illustrate yet another dysfunction unique to malformed myofibers.

It has been well established that MDX myofibers have deficits in E–C coupling, which is manifested as altered electrically elicited calcium release from the SR (Collet et al. 1999; Woods et al. 2004, 2005; Hollingworth et al. 2008). In this investigation, we confirmed a decrease in the magnitude of AP-induced SR Ca^2+^ release, in MDX myofibers with normal morphology (Fig.5). We expand these observations by demonstrating that not only do MDX malformed myofibers exhibit a further reduction in SR Ca^2+^ release when compared to both WT and MDX myofibers with normal morphology, but the branched portion of the MDX myofiber has additional deficits in SR Ca^2+^ release. It is interesting to note that Ca^2+^ transients in the branched segments of myofibers reflect the reduced Ca^2+^ release/uptake kinetics typically observed during myofiber development (Capote et al. 2005), supporting the notion that branching arises due to disruptions in the muscle growth/regeneration program (Snow and Chortkoff 1987; Tamaki et al. 1993; Head 2012). These alterations occurred without any detectable differences in resting [Ca^2+^] between WT and normal or malformed MDX myofibers (Lovering et al. 2009). Since E–C coupling deficits are thought to play a role in decreased muscle-specific force in MDX myofibers (Woods et al. 2004), and we have shown the altered morphology significantly affects E–C coupling, there appears to be an association between the overall decreased muscle function in adult MDX muscle and the high prevalence of abnormal myofiber morphology.

It seems clear at this point that malformed myofibers contribute to the dystrophic process (Lovering et al. 2009; Friedrich et al. 2010; Chan and Head 2011; Head 2012). From a structural point alone, malformed myofibers of MDX muscle could contribute to susceptibility to injury through an altered relationship between whole muscle length (*L*_0_) and muscle myofiber length (*L*_f_). If muscles of MDX mice contain myofibers that are relatively shorter than controls (e.g., branched segments of malformed myofibers), the myofibers would be subjected to greater relative strains and consequently damage more than controls (Brooks et al. 1995). The complex mechanical association of malformed myofibers with their surrounding environment remains to be elucidated. In addition to the obvious structural changes of myofiber morphology, more and more evidence indicates that these MDX malformed myofibers are further impaired than MDX myofibers with normal morphology (Lovering et al. 2009; Head 2010; Chan and Head 2011; Buttgereit et al. 2013).

The dystrophic phenotype is characterized by a decrease in the contractile force per unit area (DelloRusso et al. 2001; Lynch et al. 2001), often termed muscle specific force. A portion of this force decrement could be due to the loss in the mechanical linkage (e.g., the absence of dystrophin causing a loss of force transmission from the underlying contractile apparatus to the extracellular matrix)(Bloch and Gonzalez-Serratos 2003) or changes in E–C coupling processes (Collet et al. 1999; Woods et al. 2004, 2005; Hollingworth et al. 2008). Some reports suggest a decrease in contractile filament function (Lowe et al. 2006), while others do not (Quinlan et al. 1992). It is likely that a complex interaction between both the mechanical and signaling pathways contributes to the initiation and/or progression of the dystrophic process (Hayes and Williams 1998; Batchelor and Winder 2006).

The impact of malformed myofibers on muscle function is not fully defined. In two previous studies (Head et al. 1992; Chan et al. 2007), the occurrence of malformed myofibers in the EDL muscle was correlated with the magnitude of force loss following lengthening contractions. This result is appealing, as it coincides with the finding from Grange et al. (2002) that muscle from younger MDX mice (which has fewer malformed myofibers) is less prone to contractile-induced damage compared to muscle from older MDX mice. Furthermore, this same report by Grange et al. identified a decrease in muscle-specific force in adult muscle, but not in young muscle. Taken together, these studies support a hypothesis in which malformed myofibers contribute to damage susceptibility as well as the reduction in contractility of MDX muscle.

What cellular adaptive changes could explain the modifications seen on the AP and AP-induced Ca^2+^ transients from MDX malformed myofibers? The AP depolarization in skeletal muscle is mediated by the opening of voltage-gated Na^+^ channels and the repolarizing phase is due in part to the inactivation of the voltage-gated Na^+^ channels and the opening of multiple K^+^ channels (both voltage-dependent and independent) and Cl- channels (Jurkat-Rott et al. 2006). Here, we found that malformed MDX myofibers exhibit abnormal AP time to peak and width. Given the involvement of multiple ion channels in the sculpting of the skeletal muscle AP (Jurkat-Rott et al. 2006), it is likely that changes in the expression and/or function of one or more ion channel population could account for the observed effects at the level of AP in the malformed MDX myofibers. Early biophysical studies ruled out the possibility of the involvement of functional deficits in sarcolemmal voltage-gated Na^+^ channels and delayed rectifier K^+^ channels in MDX muscle (Mathes et al. 1991; Hocherman and Bezanilla 1996; Allard 2006). We hypothesize that changes in the expression of fast type-A voltage-gated potassium channel (such as KCNC4, also known as Kv3.4) could modify the AP properties of skeletal muscle myofibers (Vullhorst et al. 1998; Abbott et al. 1999; Rudy and McBain 2001). A role for K^+^ channels composed of Kv3.4 subunits in skeletal muscle function and disease has been considered likely because of its high expression in this tissue (Vullhorst et al. 1998; Abbott et al. 1999). It is imperative to continue electrophysiological studies to explore the presence and potential role of Kv3.4 in AP properties of malformed MDX myofibers.

While there is still some controversy regarding Ca^2+^ homeostasis in MDX myofibers, our previous report supports the finding of no difference in [Ca^2+^] at rest between control and MDX myofibers, despite significant alterations in electrically evoked Ca^2+^ transients and osmotically induced Ca^2+^ spark activity (Lovering et al. 2009). Several proposed mechanisms might explain the differences in Ca^2+^ handling found between WT and MDX myofibers. Alterations in the expression levels of proteins involved in E–C coupling (Dowling et al. 2004), changes in the ability of the myofibers to buffer/remove Ca^2+^ (Dowling et al. 2004), changes in function of membrane channels (Yeung et al. 2005), and differences in levels of ROS (Wozniak and Anderson 2008). It is possible that one or all contribute to the differences in Ca^2+^ signaling seen when comparing MDX-normal and MDX-branched myofibers, as well as when comparing the trunk and branch of a single malformed MDX myofiber. Regardless of the underlying mechanism(s), our data reveal that the amplitude of the AP-evoked Ca^2+^ transient is significantly reduced in the branched portion of the MDX malformed myofiber. This could imply that the branched segment may experience a different global Ca^2+^ signal (Berridge 1997, 2012). Given the critical role of Ca^2+^ signals in the many aspects of muscle function (i.e., contraction, gene expression, metabolism) (Berchtold et al. 2000), remodeling of the global Ca^2+^ signal and the consequent generation of inappropriate responses could dramatically affect the overall function of the branched segment of the myofibers, contributing to the progressive myopathy seen in DMD.

Muscle lacking dystrophin generates less force and is more susceptible to injury, at least in fast-twitch muscles (DelloRusso et al. 2001). The dystrophic phenotype might be a direct result of the missing protein, for example, membrane instability of the myofibers resulting in poor force transmission and/or cell death (Petrof et al. 1993). Alternatively, the dystrophic phenotype might be an indirect result, such as absence of associated proteins (Ohlendieck and Campbell 1991), changes in ion channel function (Yeung et al. 2004), or alterations in cell signaling (Thomas et al. 1998). Our findings support the additional possibility that changes in myofiber morphology may contribute to a decrease in muscle function as well as an apparent increased susceptibility to damage by demonstrating alterations on AP temporal properties, decreased AP-induced global Ca^2+^ signals in MDX fibers, and significant asymmetries in the amplitude of AP-induced Ca^2+^ signals in branched segments of malformed myofibers as well as an increase in the susceptibility to mechanical stress. We and others (Head et al. 1990; Chan et al. 2007) have shown that the architecture of dystrophic muscle myofibers becomes more abnormal with age, which may explain why, despite the consistent lack of dystrophin, MDX skeletal muscle generates less specific force and becomes more susceptible to damage with age (Chan et al. 2007). The finding that myofibers with abnormal morphology are functionally weak and more easily damaged (Head et al. 1990) supports this notion. The pathophysiological implications of these alterations in myofiber excitability, Ca^2+^ signaling, biomechanics and contractility as well as the underlying mechanisms, will be the next goals of future studies.

## Conflict of Interest

There are no competing interests or conflicts of interests for any authors.
